# Supplementary material for: Improving Circulation Half-Life of Therapeutic Candidate N-TIMP2 by Unfolded Peptide Extension
Source: Biomolecules. 2024 Sep 20;14(9):1187. doi: 10.3390/biom14091187 (PMC11429640; doi:10.3390/biom14091187)
Supplement: Supplementary file 1 [file biomolecules-14-01187-s001.zip › biomolecules-3112262-supplementary.pdf]

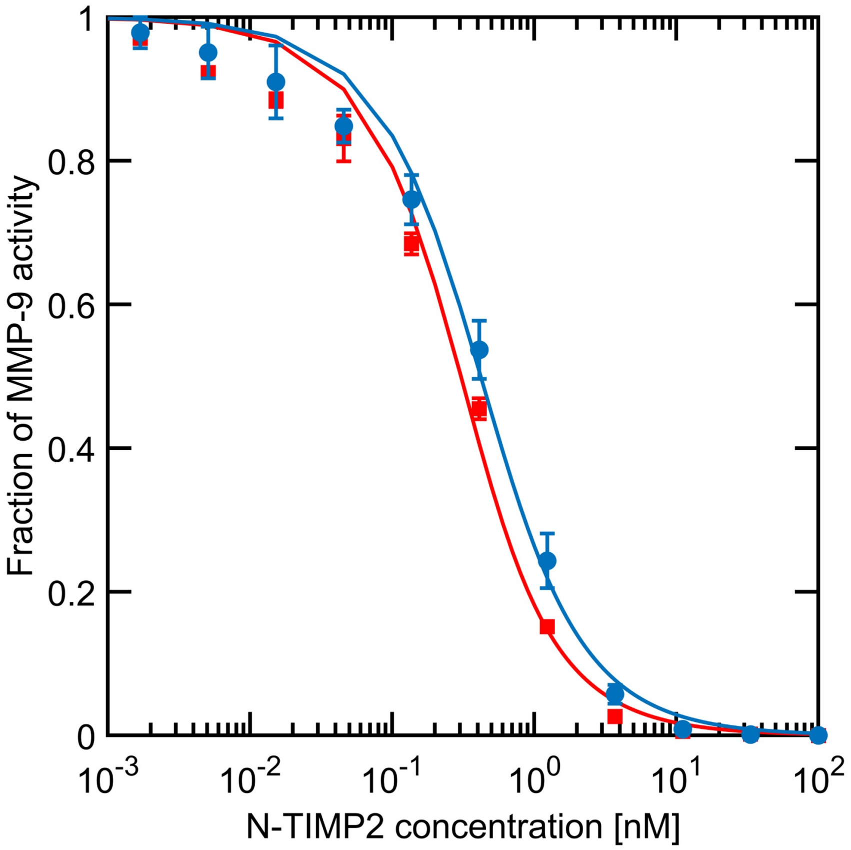

Figure S1: Inhibitory activity of N-TIMP2 with an internal FLAG-tag. Inhibitory activity of WT N-TIMP2 (red) and N-TIMP2 containing the internal FLAG-tag (blue) against MMP-9. Fraction of MMP-9 activity is plotted vs. concentration of added N-TIMP2-based inhibitor. The data were fitted to eq. 1 to obtain K_i^{app}.

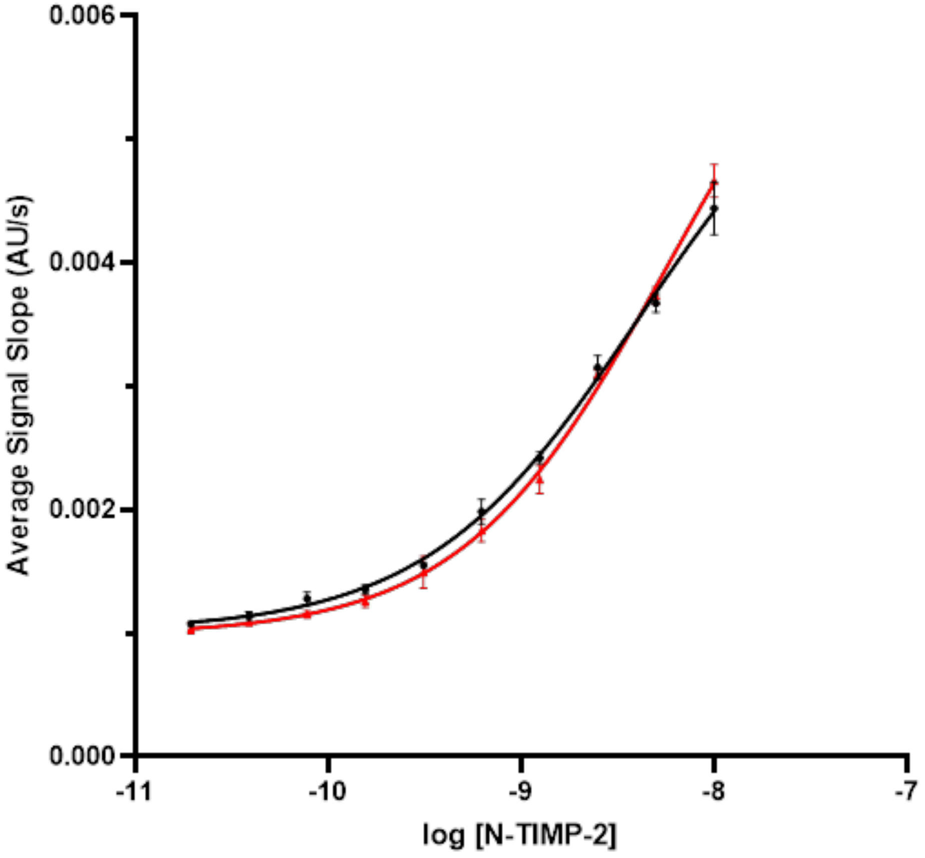

Figure S2: Standard curve plot from N-TIMP-2-PAT$_{200}$. Shown are standard curves from two different ELISA plates, where the y-axis represents the average slope of the linear portion of the 655nm absorbance rate curves, while the x-axis represents the logarithm of the known N-TIMP-2-PAT$_{200}$ concentration from pure protein sample. Each point represents triplicate wells for each concentration, with the error bars indicating the SEM.

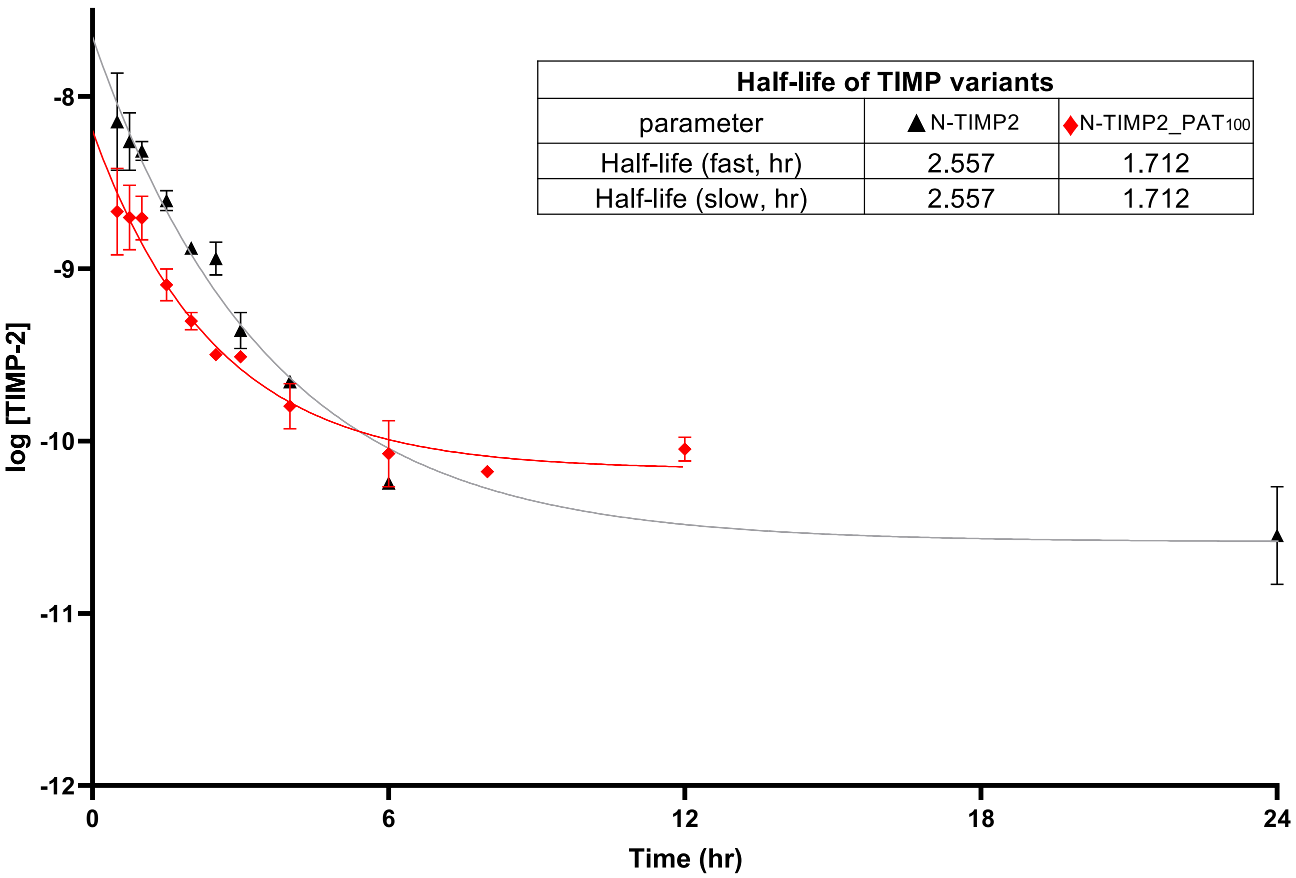

Figure S3: Elimination half-life of N-TIMP2 and N-TIMP2_PAT$_{100}$ variant. Plot of average ± SEM (n=3) of log10 N-TIMP2 concentration vs time, modeled with a 2-phase decay with least-squares nonlinear fit for half-life determination. Statistical significance assessed via a sum-of-squares F test where H0: K_{slow} N-TIMP2_PAT$_{100}$ = K_{slow} N-TIMP2; Ha: K_{slow} N-TIMP2_PAT$_{100}$ $\neq$ K_{slow} N-TIMP2. $\alpha=0.05$, $p=0.9927$.
